# CHEOPS trial: a GINECO group randomized phase II assessing addition of a non-steroidal aromatase inhibitor to oral vinorelbine in pre-treated metastatic breast cancer patients

**DOI:** 10.1007/s12282-022-01426-1

**Published:** 2023-01-05

**Authors:** Caroline Bailleux, Antoine Arnaud, Jean-Sébastien Frenel, Sylvie Chabaud, Thomas Bachelot, Benoît You, Laëtitia Stefani, Claire Garnier Tixidre, Hélène Simon, Dominique Beal-Ardisson, Jean-Philippe Jacquin, Francesco Del Piano, Alain Lortholary, Claudiu Cornea, Charlotte Greilsamer, Rémy Largillier, Fabien Brocard, Eric Legouffe, Mustapha Atlassi, Anne-Claire Hardy-Bessard, Pierre-Etienne Heudel

**Affiliations:** 1 Department of Medical Oncology, Centre Léon Bérard, 28 Rue Laennec, 69008 Lyon, France; 2 Institut du Cancer Avignon-Provence, 250 Chemin de Baigne-Pieds, CS 800005, 84918 Avignon, France; 3 Institut de Cancérologie de L’Ouest, Centre René Gauducheau, Boulevard Jacques Monod, 44805 Saint Herblain, France; 4 Centre Hospitalier Lyon Sud, 165 Chemin du Grand Revoyet, 69495 Pierre Bénite, France; 5 Centre Hospitalier Annecy Genevois, 1 Avenue de l’Hôpital, BP 90074, 74374 Pringy, France; 6 Centre Hospitalier Mutualiste de Grenoble, 8 Rue Docteur Calmette, 38028 Grenoble, France; 7 Hôpital Morvan, CHU de Brest, 5 Avenue Foch, 29200 Brest, France; 8 Hôpital Privé Jean Mermoz, 55 Avenue Jean Mermoz, BP 7322, 69373 Lyon, France; 9 Institut de Cancérologie de La Loire Lucien Neuwirth, 108 Bis Avenue Albert Raimond, 42271 Saint Priest en Jarez, France; 10 Hôpitaux du Léman, 3 Avenue Dame, BP 526, 74203 Thonon Les Bains, France; 11 Hôpital Privé du Confluent, 2-4 Rue Eric Tabarly, BP 20215, 44202 Nantes, France; 12 Centre Hospitalier Jean-Bernard, 114 Avenue Desandrouins, BP 479, 59322 Valenciennes, France; 13 Centre Hospitalier Départemental Vendée Les Oudairies, Boulevard Stéphane Moreau, 85925 La Roche Sur Yon, France; 14 Centre Azuréen de Cancérologie, 1 Place du Docteur Jean-Luc Broquerie, 06250 Mougins, France; 15 ORACLE–Centre d’Oncologie de Gentilly, 2 Rue Marie Marvingt, 54000 Gentilly, France; 16 Institut de Cancérologie du Gard Centre ONCOGARD, Rue du Professeur Henri Pujol, 30900 Nimes, France; 17 Centre Hospitalier Le Mans, 194 Avenue Rubillard, 72000 Le Mans, France; 18 Centre CARIO-HPCA, 10 Rue François Jacob, 22190 Plérin, France

**Keywords:** Randomized phase II, Aromatase inhibitor, Oral vinorelbine, Metastatic breast cancer

## Abstract

**Background:**

The objective of the CHEOPS trial was to assess the benefit of adding aromatase inhibitor (AI) to metronomic chemotherapy, oral vinorelbine, 50 mg, three times a week for pre-treated, HR + /HER2- metastatic breast cancer patients.

**Methods:**

In this multicentric phase II study, patients had to have progressed on AI and one or two lines of chemotherapy. They were randomized between oral vinorelbine (Arm A) and oral vinorelbine with non-steroidal AI (Arm B).

**Results:**

121 patients were included, 61 patients in Arm A and 60 patients in Arm B. The median age was 68 years. 109 patients had visceral metastases. They all had previously received an AI. The study had been prematurely stopped following the third death due to febrile neutropenia. Median PFS trend was found to be different with 2.3 months and 3.7 months in Arm A and Arm B, respectively (HR 0.73, 95%CI 0.50–1.06, *p* value = 0.0929). No statistical difference was shown in OS and better tumor response. 56 serious adverse events corresponding to 25 patients (21%) were reported (respectively, 12 (20%) versus 13 (22%) for arms A and B) (NS).

**Conclusion:**

The addition of AI to oral vinorelbine over oral vinorelbine alone in aromatase inhibitor-resistant metastatic breast cancer was associated with a non-significant improvement of PFS. Several unexpected serious adverse events were reported. Metronomic oral vinorelbine schedule, at 50 mg three times a week, requires close biological monitoring. The question of hormonal treatment and chemotherapy combination remains open.

## Introduction

Through their mitogenic effects via the estrogen-receptor alpha (ERα), estrogens play a fundamental role in the carcinogenesis process. Blocking estrogenic signaling is therefore the basic principle of hormone therapy for the treatment of hormone receptor-positive (HR +) breast cancer. Despite the undeniable efficacy of molecules used in endocrine therapy, many tumors have intrinsic or acquired resistance, despite their positive tumor status for ERα. It is nevertheless interesting to note that some clinical studies have shown that 43% and 30% of patients who have relapsed, respectively, on tamoxifen or aromatase inhibitor will respond to fulvestrant treatment, indicating the interest of continuing endocrine therapy beyond neoplastic progression [1, 2].

Systemic chemotherapy with cytotoxic agents remains a standard treatment for metastatic cancer. "Metronomic" chemotherapy is a repeated reduced-dose chemotherapy treatment administered daily for antineoplastic purposes. These effects involve anti-angiogenic impact, interference with immune response and conventional cytotoxic activity [3]. The impact of metronomic chemotherapy on angiogenesis would be explained by the inhibition of the mobilization of endothelial cell progenitors and/or the activation of apoptosis by endothelial cells [4]. There may also be a reactivation of the immune system through a reduction in the number of regulatory T cells, a decrease in their inhibitory function of T and NK lymphocyte activity and a maturation of dendritic cells, thus stimulating the antitumor immune response [5].

Metronomic chemotherapy was initially evaluated in 64 metastatic breast cancer patients by an Italian Phase II study. The treatment included methotrexate and cyclophosphamide and interesting results were reported, with 20% response rate, a 30% clinical benefit rate and no significant toxicity [6]. Regarding metronomic administration of vinorelbine, several regimens with multiple dosages were tested in metastatic breast cancer treatment. Oral vinorelbine according to a 50 mg metronomic regimen, three times a week continuously, was evaluated as monotherapy in a phase I study with pharmacokinetic data [7] and in combination with bevacizumab [8]. These studies showed that administration of 50 mg of oral vinorelbine three times a week was feasible and well tolerated with an interesting clinical benefit in advanced refractory cancers.

The combination of endocrine therapy and metronomic chemotherapy with vinorelbine could therefore be of interest for HR + /HER2− (Hormone receptor positive, human epidermal growth factor receptor 2 negative) breast cancers in a metastatic hormone-resistance setting.

We hypothesized that maintaining HR-targeted therapy after progression in combination with chemotherapy may improve disease control. The CHEOPS study aims to confirm the clinical benefit of a combination of an anti-aromatase and metronomic chemotherapy treatment, oral vinorelbine (OV), 50 mg, three times per week for AI pre-treated, HR + /HER2− metastatic breast cancer patients. It would have the theoretical advantage of being well tolerated and more effective than chemotherapy alone even after an anti-aromatase therapy.

## Materials and methods

### Population

In this national, multicentric, randomized, open-label phase II study, patients had to have progressed on endocrine therapy and one or two lines of chemotherapy for HR + /HER2− metastatic breast cancer. Inclusion criteria were: age ≥ 50 years, post-menopausal woman, ECOG performance status (PS) 0, 1 or 2, adequate biological function (polynuclear neutrophils ≥ 1,5.10^9^/L; platelets ≥ 100.10^9^/L; creatinine clearance ≥ 30 mL/min; total bilirubin ≤ 1.5 times the upper limit of normal (×ULN); alkaline phosphatases ≤ 2.5 ×ULN; ALAT, ASAT ≤ 1.5 ×ULN in the absence of liver metastases or ≤ 3 ×ULN in the presence of liver metastases), histologically proven breast cancer, progesterone and /or estrogen receptors positive, HER2 negative on primary tumor, patient taking hormonotherapy, in progression, already treated by at least one line of anti-aromatase non-steroidal therapy and by at least one line of chemotherapy and no accessibility to surgical treatment; patient having to begin a second or third line of chemotherapy, no previous treatment containing vinorelbine, presence of one or several measurable(s) or assessable(s) metastatic lesion(s) according to RECIST 1.1; patient with a life expectancy greater than 3 months, without non-irradiated cerebral or symptomatic metastasis, without symptomatic pulmonary carcinomatosis lymphangitis, without known allergies to anastrozole, letrozole or vinorelbine; patient with informed consent signed before enrollment and affiliation to a social security scheme.

### Ethics

This study was approved by an ethics committee (Independent Protection Committee 15/026, No. EudraCT 2015–000,401-39) and registered on Clinicantrials.gov as NCT02585388. Patients gave informed consent at the first consultation. The Independent Data Monitoring Committee (IDMC) analyzed interim efficacy data and study-related adverse events.

### Study design

Patients were randomized between oral vinorelbine metronomic three times a week (Mondays, Wednesdays, Fridays or Thursdays, Tuesdays, Saturdays) at 50 mg per day in combination with non-steroidal aromatase inhibitor, letrozole 2,5 mg every day or anastrozole 1 mg every day (Arm B) and oral vinorelbine alone (Arm A). Treatments were taken orally until progression of disease or toxicity. Dose adjustment of oral vinorelbine was possible in case of toxicity. Primary outcome measure was progression-free survival (PFS) evaluated every 8 weeks. Secondary outcome measures were evaluation of partial and complete response rate by RECIST 1.1, duration of response, clinical benefit after 24 weeks of treatment, overall survival, toxicity according to criteria NCI CTAEv4.03 evaluated every 4 weeks and health-related quality of life evaluated every 8 weeks with EORTC QLQ-C30 questionnaire.

### End points

Progression-free survival (PFS) was defined as time from inclusion to first documentation of objective disease progression or death due to any cause or until the date of the last news (censored data). Evaluation of partial and complete response rate was performed by RECIST 1.1 in each arm. Duration of response was defined as the time from first met for complete or partial response (CR/PR) (whichever is first recorded) until the first date that recurrent or progressive disease which was objectively documented and calculated only in patients with a response to treatment (CR/PR). Clinical benefit is defined by the rate of complete response, the rate of partial response and the stability of lesions at 24 weeks according to criteria RECIST 1.1. Overall survival (OS) was defined as time from inclusion to death due to any cause. Tolerance of the treatment was based on adverse events occurrence according to criteria NCI CTCAEv4.03.

### Sample size

To show an increase of median PFS (from 3.5 to 5.5 months, HR 0.636), with unilateral alpha = 5% and power = 80%, 130 evaluable patients were needed for 121 events at the time of the final analysis. Randomization, to a 1:1 ratio, was stratified according to the inclusion center and the number of lines of chemotherapy (second versus third line). All efficacy analyses were conducted on the intending to treat (ITT) population. Two safety interim analyses were scheduled.

### Statistics

Qualitative parameters were described in each arm and then compared between the two arms by the Chi^2^ test or the Fisher exact test depending on the number of patients. Survival parameters (PFS and OS) were estimated using Kaplan–Meier method and described in terms of median associated with two-sided 95% confidence intervals in each arm. Survival distributions were compared between the arms using a log-rank test, supported by a Cox regression. The rates of patients with toxicity, toxicity grade ≥ 3, toxic death, or a serious adverse event will be described by treatment arm and compared according to a Chi^2^ test or a Fisher test according to the number of patients.

The analyses were performed using SAS software version 9.3 (SAS Institute, Cary, NC).

## Results

### Population

**Fig. 1:**
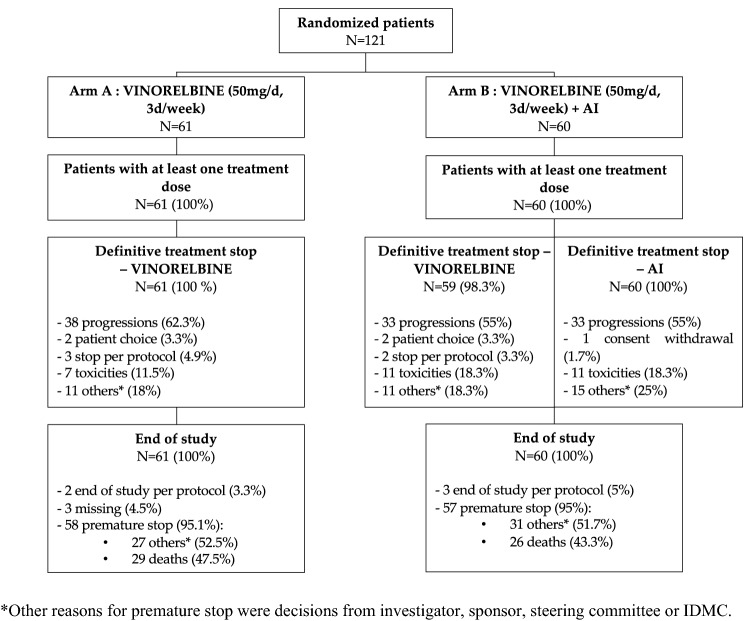
Flowchart. AI: aromatase inhibitor. *Other reasons for premature stop were decisions from investigator, sponsor, steering committee or IDMC

Between October 2015 and May 2017, 27 cancer centers participated in the CHEOPS trial. The participating centers was diverse, with private, public and dedicated cancer centers. Overall, 121 patients were included and randomized: 61 patients received oral vinorelbine (Arm A) and 60 patients a combination of oral vinorelbine and aromatase inhibitor (Arm B) (Fig. 1). The median age was 68 years (range 49–87).  The performance status was PS0, PS1 and PS2 for 50 patients (41.7%), 64 patients (53.3%) and 6 patients (5%), respectively. 24 patients (20%) were metastatic at the time of diagnosis. Delay since metastatic diagnosis was 3.2 years (range 0—16.9). 109 patients (90%) had visceral metastases. They all had previously received an aromatase inhibitor; nine patients had received it only in the adjuvant setting. Patients were randomized after one line of chemotherapy (*N* = 66, 54.5%) or two lines of chemotherapy (*N* = 55, 45.5%). Seven patients (Arm A: 4; Arm B: 3) had previously received anti-CDK4/6 therapy. Patient features were well balanced between the two treatment arms ( Table 1).

**Table 1:** Patient, tumor and treatment characteristics

	Randomization arm				All patients	
	A: vinorelbine		B: vinorelbine			
			+ aromatase inhibitor			
	*N* = 61		*N* = 60		*N* = 121	
Age						
Median (min; max)(years)	67.8 (48.6; 87.3)		66.9 (50.0; 80.4)		67.7 (48.6; 87.3)	
Histologic type						
Ductal carcinoma	43	(72.9%)	44	(75.9%)	87	(74.4%)
Lobular carcinoma	10	(16.9%)	11	(19.0%)	21	(17.9%)
Mixed carcinoma	4	(6.8%)	1	(1.7%)	5	(4.3%)
Others	2	(3.4%)	2	(3.4%)	4	(3.5%)
SBR grade						
I	6	(10.0%)	7	(11.7%)	13	(10.8%)
II	33	(55.0%)	39	(65.0%)	72	(60.0%)
III	13	(21.7%)	8	(13.3%)	21	(17.5%)
Unknown	8	(13.3%)	6	(10.0%)	14	(11.7%)
Estrogen receptor						
Negative	2	(3.3%)	1	(1.7%)	3	(2.5%)
Positive	56	(91.8%)	54	(91.5%)	110	(91.7%)
Unknown	3	(4.9%)	4	(6.8%)	7	(5.8%)
Progesterone receptor						
Negative	15	(25.0%)	15	(25.4%)	30	(25.2%)
Positive	42	(70.0%)	40	(67.8%)	82	(68.9%)
Unknown	3	(5.0%)	4	(6.8%)	7	(5.9%)
HER 2/IHC						
0	37	(72.5%)	38	(74.5%)	75	(73.5%)
1 +	12	(23.5%)	11	(21.6%)	23	(22.5%)
2 +	2	(3.9%)	2	(3.9%)	4	(3.9%)
Delay since metastatic diagnosis						
Median (min; max) (years)	2.9 (0.0; 12.5)		3.4 (0.1; 16.9)		3.2 (0.0; 16.9)	
Metastatic sites						
Bone metastasis only	8	(13.1%)	4	(6.7%)	12	(9.9%)
Liver metastasis	28	(46.7%)	36	(60.0%)	64	(53.3%)
CNS metastasis	0	(0.0%)	2	(3.3%)	2	(1.7%)
Number of previous lines for metastatic disease						
1	8	(13.1%)	8	(13.3%)	16	(13.2%)
2	24	(39.3%)	16	(26.7%)	40	(33.1%)
3	15	(24.6%)	13	(21.7%)	28	(23.1%)
≥ 4	14	(23.0%)	23	(38.3%)	37	(30.6%)
Number of prior chemotherapy line(s)						
1	34	(55.7%)	32	(53.3%)	66	(54.5%)
2	27	(44.3%)	28	(46.7%)	55	(45.5%)
Previous hormone therapy for metastatic diagnosis						
No	5	(8.2%)	4	(6.7%)	9	(7.4%)
Yes	56	(91.8%)	56	(93.3%)	112	(92.6%)
Main details of previous treatments received						
Anthracycline	13	(21.1%)	9	(15.2%)	22	(18%)
Taxane	49	(80.4%)	50	(83.3%)	99	(81.8%)
Capecitabine	28	(45.9%)	27	(45.0%)	55	(45.5%)
Fulvestrant	29	(47.5%)	30	(50.0%)	59	(48.7%)
Letrozole	32	(52.5%)	34	(56.7%)	66	(54.5%)
Anastrozole	15	(24.6%)	13	(21.7%)	28	(23.1%)
Exemestane	28	(45.9%)	38	(63.3%)	66	(54.5%)
CDK 4/6 inhibitor	5	(8.2%)	5	(8.4%)	10	(8.2%)

Table does not include missing data; no significant difference found with Fisher's test and Chi^2^ test. *SBR* Scarff–Bloom–Richardson grade, *HER 2/IHC* Immunohistochemistry estimation of human epidermal growth factor receptor 2 expression, *CNS* central nervous system, *CDK* cyclin-dependent kinase

### Treatment

The causes of treatment discontinuation are summarized in Fig. 1. The median duration of treatment with vinorelbine was 1.8 months (0.0–15.6) and was similar between the two arms with 1.8 months (0.0–13.3) and 1.9 months (0.3–15.6) in Arm A and B, respectively. Nine patients (7%) had a dose reduction, seven of them for hematological or digestive toxicities. Thirty-five patients (29%) temporarily stopped treatment with vinorelbine, 26 of them due to toxicity. 120 patients (99.2%) definitively discontinued treatment with vinorelbine. For 71 patients (58.7%), the cause of discontinuation of treatment was progression. Other causes of permanent discontinuation of vinorelbine are: patient choice (*N* = 4), protocol discontinuation (*N* = 5, patient reached 18 months of post-treatment follow-up), toxicity (*N* = 18) and other cause (*N* = 22, mainly at the request of the sponsor following the decision to discontinue the study). Regarding endocrine therapy, 58% of patients were treated with letrozole and 42% with anastrozole.

### Primary end point: progression-free survival (PFS)

**Fig. 2:**
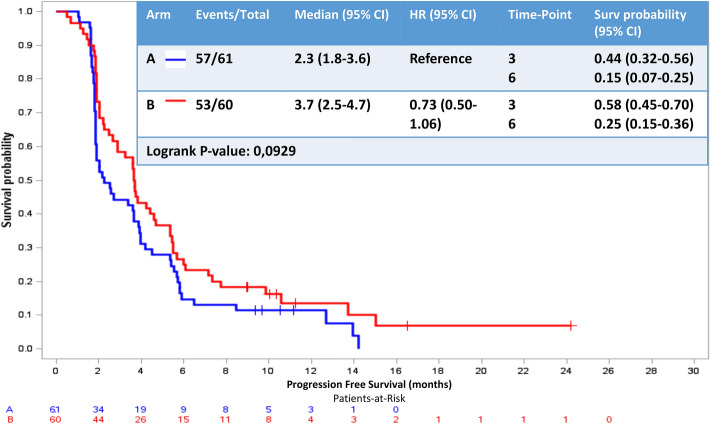
Progression-free survival/primary end point. *HR* hazard ratio, *95% CI* 95% confidence interval, time point: 3 months and 6 months. Progression-free survival not reached statistically significance with log-rank *P* value > 0.05

Median PFS was 2.3 months (95% CI 1.8–3.6) and 3.7 months (95% IC 2.5–4.7) in Arm A and Arm B, respectively (HR 0.73, 95% CI 0.50–1.06, log rank *P* value = 0.0929) (Fig. 2). The  oral vinorelbine–endocrine therapy combination was more effective than oral vinorelbine alone even if statistical significance was not reached.

### Secondary end point

**Fig. 3:**
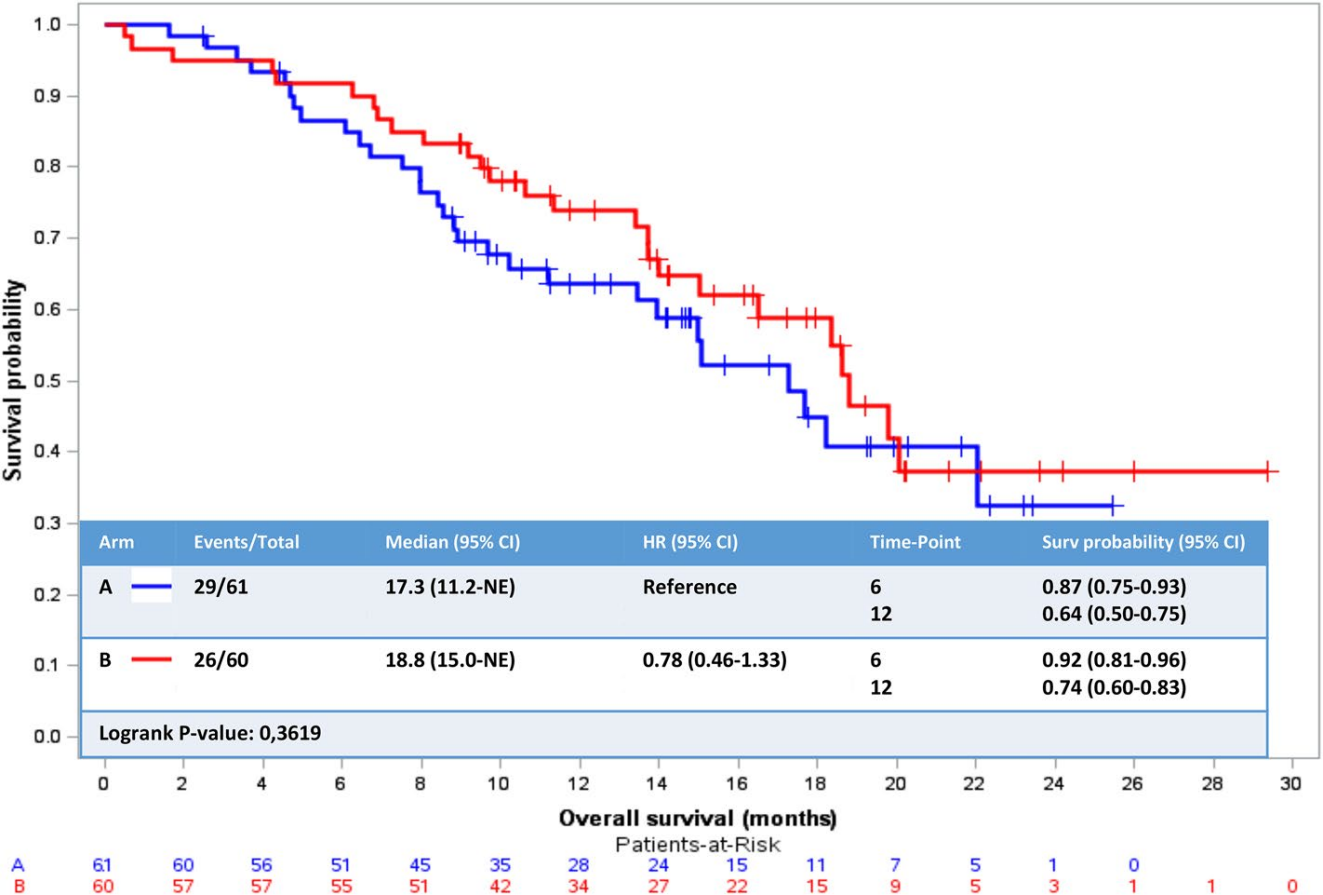
Overall survival/secondary end point. *Arm A* oral vinorelbine monotherapy, *Arm B* oral vinorelbine + aromatase inhibitor. *HR* hazard ratio, *95% CI* 95% confidence interval; time point: 6 months and 12 months. Overall survival not reached statistical significance with log-rank *P* value > 0.05

Nine patients (5 in Arm A (10%) and 4 in Arm B (8%)) had an objective response to treatment (complete response or partial response). No statistically significant difference was found between the two arms. The median response duration was 3.7 months and 2.8 months in Arm A and Arm B, respectively. No difference was demonstrated between the two arms. Concerning best tumor response, only one complete response was observed in Arm A and none in Arm B. Four patients (7.7%) in each arm obtained a partial response and 19 patients (36.5%) and 30 patients (57.7%) had a stable disease, respectively, in Arm A and Arm B. No statistical difference was found in terms of better tumor response (Fisher exact test, *p* = 0.122) (Table 2). At 24 weeks, 15 patients (24.6%) and 17 patients (28.3%) were non-progressive in Arm A and Arm B, respectively. Concerning overall survival, with a median follow-up of 16.5 months (2.5–29.4 months), no statistical difference was shown in OS with a median of 17.3 months (95% CI 11.2-NE) and 18.8 months (95% CI 15.0-NE) in Arm A and Arm B, respectively (HR 0.78, 95% CI 0.46–1.33, log-rank *P* value = 0.3619) (Fig. 3). Among functional scales, comparing the time of diagnosis and at the end of treatment, only three scores were statistically different between the arms in favor of Arm A (oral vinorelbine): physical functioning score (*p* = 0.034), emotional functioning score (*p* = 0.017) and social functioning score (*p* = 0.045). No difference was found for global quality of life score, role functioning score, financial impairment scale and cognitive functioning score. Among symptom scales (fatigue, nausea–vomiting, pain, dyspnea, insomnia, loss of appetite, diarrhea and constipation), only fatigue (*p* = 0.007), insomnia (0.003) and loss of appetite (*p* < 0.001) were statistically different in favor of Arm B (oral vinorelbine + AI) (Appendix Table 4).

**Table 2:** Best tumor response

	Randomization arm				All patients		Test Fisher exact
	A: vinorelbine		B: vinorelbine				
			+ aromatase inhibitor				
	*N* = 61		*N* = 60		*N* = 121		
Best response							*P* = 0.122
Complete response	1	(1.9%)	0	(0.0%)	1	(1.0%)	
Partial response	4	(7.7%)	4	(7.7%)	8	(7.7%)	
Stability	19	(36.5%)	30	(57.7%)	49	(47.1%)	
Progression	27	(51.9%)	18	(34.6%)	45	(43.3%)	
Not evaluable	1	(1.9%)	0	(0.0%)	1	(1.0%)	
Missing data*	9		8		17		

*17 patients did not have a radiologic reevaluation incompliance with protocol deadlines and were therefore not included in the best tumor response evaluation

**Table 3:** Main adverse events grade ≥ 3 (frequency > 10%)

	A: vinorelbine			B: vinorelbine			All patients		
				+ aromatase inhibitor					
	Subjects		Events	Subjects		Events	Subjects		Events
	*N* = 61		*N* = 165	*N* = 60		*N* = 198	*N* = 121		*N* = 363
Adverse event	40	(65.6%)	142	40	(66.7%)	173	80	(66.1%)	315
Gamma GT increase	13	(21.3%)	30	15	(25.0%)	45	28	(23.1%)	75
Neutropenia	10	(16.4%)	15	12	(20.0%)	18	22	(18.2%)	33
Arterial hypertension	7	(11.5%)	7	14	(23.3%)	25	21	(17.4%)	32
Lymphopenia	11	(18.0%)	14	10	(16.7%)	14	21	(17.4%)	28
Fatigue	10	(16.4%)	15	5	(8.3%)	6	15	(12.4%)	21

No statistically significant difference was found between the two treatment arms

### Main adverse events

Table 3 summarizes all AE grade ≥ 3 reported in at least 10% of patients. The most frequent overall adverse events are: GGT increase (73%), fatigue (67%), high blood pressure (67%), lymphopenia (66%), ASAT increase (59%), anemia (58%) and nausea (53%). At baseline, five patients (8.2%) in Arm A and four patients (6.7%) in Arm B had sensory neuropathy. Only one patient (Arm B) had motor neuropathy. During treatment, sensory neuropathy appeared for two and five additional patients in arms A and B, respectively. 81 patients (67%) had at least one grade of ≥ 3 adverse event (respectively, 40 (66%) versus 41 (68%) for arms A and B). 56 serious adverse events corresponding to 25 patients (21%) were reported (respectively, 12 (20%) versus 13 (22%) for arms A and B): 9 SAE grade 3, 18 SAE grade 4 and 8 SAE grade 5 with 14 severe cytopenia, 9 sepsis, 4 severe digestive disorders, 3 central neurological complications, 2 asthenia, 2 GGT increases and 1 severe pain. Overall occurrence and severe adverse events are detailed in Appendix Table 5. No statistically significant difference was found between the two treatment arms.

The study has been prematurely stopped upon IDMC decision following the third death due to treatment toxicity (febrile neutropenia) after 121 patients were randomized: 1 in Arm A and 2 in Arm B, secondary to febrile neutropenia. Toxic deaths following febrile neutropenia were observed. Patients were 68-year-old, 67-year-old and 80-year-old female patients, each with known diabetes and hypertension. Febrile neutropenia occurred during the first cycle at day 9 and day 10 of vinorelbine + anastrozole for those in Arm B, and at day 30 for the patient in Arm A. Death occurred on day 17 and day 19 in Arm B, and on day 40 in Arm A. Patients experienced: 1) a rapidly unfavorable evolution of sepsis respiratory distress due to *E. Coli* infection; 2) a refractory septic shock to *Pseudomonas aeruginosa* complicated with multi-organ failure; 3) craniocerebral injury following pulmonary sepsis, respectively.

## Discussion

The results of the CHEOPS study did not reach statistical significance, but showed a modest potential benefit of combining hormone therapy and metronomic chemotherapy in metastatic breast cancer HR + /HER2− pre-treated with endocrine therapy (HR 0.73, 95% CI 0.50–1.06, log-rank *P* value = 0.0929). Data are still immature due to premature termination due to much higher than expected toxicity.

The limiting factor in this study was the number of toxic deaths induced by oral vinorelbine dose and administration scheme. Indeed, adverse effects of metronomic chemotherapy are most often mild or non-existent and are generally represented by grade 1 toxicities: leukopenia, moderate neutropenia, nausea and vomiting, increased transaminases and asthenia. Serious grade 3–4 toxicities are rare [9, 10]. Several studies have confirmed that metronomic oral vinorelbine can safely be administered at doses up to 50 mg three times a week, especially in advanced breast cancer [11, 12]. Patients with recurrent metastatic breast (BC), prostate (PC) or non-small cell lung cancer (NSCLC) and adequate organ functions were randomly assigned to 30, 40 or 50 mg vinorelbine, taken orally three times a week. With maximum response duration achieved at 50 mg, adverse events were mild and negligible and did not differ between the three arms. Considering the antitumor activity and response duration, the negligible toxicity of the highest dose investigated and the lack of drug accumulation over time, the authors suggest that 50 mg given three times a week is the optimal dose for metronomic oral vinorelbine [11].

The toxic deaths observed here may be the result of poor management of oral oncology drugs at home, for example, by maintaining chemotherapy during periods of neutropenia, infection or hospitalization, despite protocol recommendations. Recommendations for close biological monitoring have been strengthened accordingly (Appendix Table 6). Serious grade 3–4 toxicities would be better managed today through better knowledge of adverse reactions and learning by mistake. The management of per os cancer drugs requires patient autonomy, training of paramedical staff and the knowledge of all health-care providers in contact with the patient. This experience shows that it is now necessary to train non-specialized staff to provide the best possible support to patients treated at home [13–15]. The other possibility is to introduce progressively oral vinorelbine with dose escalation scheme or reduce the dose of oral vinorelbine administered three times a week to minimize the risk of toxic death. Oral form of vinorelbine 70 mg/m^2^ (fractionated on days 1, 3, and 5 for 3 weeks, on and 1 week off, every 4 weeks, for a maximum of 12 cycles) has been experimented in 34 elderly metastatic breast cancer patients and an OR of 38% was reported. Neutropenic infection was evident in two patients (6%). In all instances, these complications resolved during antibiotic therapy [16]. Another study with an alternative on and off metronomic regimen, vinorelbine 30 mg (total dose), one day on and one day off, was given to 32 elderly patients with metastatic breast cancer; a 50% CB was reported, without grade 3 or 4 toxicity [17]. Adamo et al. reported the biological effect of oral metronomic vinorelbine alone or in combination with endocrine therapy in 61 post-menopausal women with untreated stage I–III HR + /HER2-negative breast cancer. Two cases (3.4%) of grade 3 adverse event, both in the oral metronomic vinorelbine alone arm, were observed after completing the 3-week treatment. One case was an acute pancreatitis and the other was an acute gastroenteritis. Overall, no discontinuations due to toxicity was observed. However, the study population was treatment naive, whereas patients in the CHEOPS trial were already at an advanced stage of their metastatic disease (average of 3.2 years since diagnosis of metastatic disease) and had already received one or two lines of chemotherapy [18].

Other combinations may then be possible such as capecitabine and endocrine therapy or oral cyclophosphamide and endocrine therapy. Some studies evaluating combinations were carried out in the 1980s. These randomized trials compared chemotherapy most often with CMF (cyclophosphamide, methotrexate and 5 fluorouracil) as monotherapy with the same chemotherapy in combination with tamoxifen. These different studies showed significant benefit in terms of neoplastic response rate (74% versus 51%, respectively; P < 0.01; 75% versus 49% p = 0.0001) and progression-free survival. However, there was no significant difference in terms of overall survival (111 weeks versus 78 weeks *p* = 0.25 [19]; 24 months versus 19 months *p* = 0.07 [20, 21]), mainly due to side effects including thromboembolic effects induced by tamoxifen. However, in patients with metastatic breast cancer, aromatase inhibitor (AI) has been shown to be superior in terms of tamoxifen survival and significantly reduce thromboembolic complications [22]. A randomized phase II clinical trial evaluated the combination of letrozole and cyclophosphamide (50 mg/d for 6 months) compared to letrozole alone in 114 patients with hormone-sensitive metastatic breast cancer. The authors concluded that metronomic cyclophosphamide associated with hormone therapy was beneficial with an objective response rate of 87% in the combination arm versus 71% in the letrozole arm alone [23]. However, these trials mainly excluded patients resistant to concomitant endocrine therapy. In contrast, all patients enrolled in the CHEOPS trial were resistant to AI. This difference may be associated with the small benefit of adding AI to chemotherapy reported here. For HR + /HER2− pre-treated metastatic breast cancer, efficacy of endocrine therapy in combination with chemotherapy remains an open question.

The underlying question is then the interest of maintaining endocrine therapy throughout the treatment, including successive chemotherapy lines. To finish, maintenance endocrine therapy (MET) after chemotherapy could be another way to combine endocrine therapy and chemotherapy. Sutherland et al. discussed four trials addressing the question of whether there is a benefit from introducing endocrine therapy following chemotherapy for metastatic breast cancer [24]. Berrutti et al. investigated the factors influencing response rate and overall survival in 207 MBC patients responding to first-line chemotherapy with epirubicin administration, followed or not by MET. Patients receiving MET survived significantly longer than those submitted to observation in univariate and multivariate analysis [25]. In a phase III randomized trial, Kloke et al. investigated the use of medroxyprogesterone acetate (MPA) maintenance treatment in 90 advanced breast cancer patients with a disease controlled after six cycles of induction chemotherapy. A longer median time to progression (TTP) was reported in the MET arm compared to the observation arm (4.9 months versus 3.0 months), but no difference in OS was observed (17.4 months versus 18.0 months) [26]. Montemmuro et al. retrospectively analyzed the effect of MET after high-dose chemotherapy with hematopoietic progenitor cell transplant (HDCT) on the progression-free survival (PFS) on 109 patients with hormone-dependent MBC who remained progression free for at least 4 months after HDCT. Of these patients, 55 were non-randomly submitted to MET. In multivariate analysis, MET appeared to be a significant factor with improvement of PFS with MET (HR 0.58; 95% CI: 0.362–0.931) [27]. Finally, Dufresne et al. retrospectively identified factors which influence PFS and OS after the first line of chemotherapy in 560 patients with HR + MBC. Administration of MET was shown to improve both PFS (16.3 versus 7.8 months; *p* < 0.001) and OS (48.1 versus 30 months; *p* < 0.0001) in multivariate analysis [28]. When chemotherapy for MBC was discontinued due to toxicity, in the absence of progression, the use of ET, with its relatively low toxicity, is a reasonable option, although this approach has not been assessed in randomized trials.

Concerning prior treatment, only a minority of patients had previously received anti-CDK4/6 therapy. This treatment is now the gold standard for first-line treatment of HR + metastatic breast cancer. The results of the CHEOPS trial are therefore not applicable to current daily practice. However, these results do provide a proof of concept on a trend toward an improvement of outcomes with a combination of endocrine therapy and chemotherapy compared to chemotherapy alone. This concept should persist after exposure to anti-CDK4/6.

## Conclusions

The addition of aromatase inhibitor to oral vinorelbine over oral vinorelbine alone in aromatase inhibitor resistant metastatic breast cancer was associated with a non-significant improvement of PFS. The study has been prematurely stopped due to treatment toxicity. Several unexpected SAEs were reported. Metronomic oral vinorelbine schedule, at 50 mg three times a week, requires close biological monitoring. The question of hormonal treatment and chemotherapy combination remains open.

## Data Availability

The data that support the findings of this study are available from the corresponding author upon reasonable request.

## Appendix

See appendix Tables 4, 5, 6

**Table 4:** Quality of life analysis

	Randomization arm				All patients		Test
	A: vinorelbine		B: vinorelbine				
			+ aromatase inhibitor				
	*N* = 38		*N* = 43		*N* = 81		Fisher exact
Functional scales							
Evolution of the global QL score							*p* = 0.068
Worsened	10	(26.3%)	22	(51.2%)	32	(39.5%)	
Stable	18	(47.4%)	15	(34.9%)	33	(40.7%)	
Improved	10	(26.3%)	6	(14.0%)	16	(19.8%)	
Evolution of the physical functioning score							*p* = 0.034
Worsened	8	(21.1%)	19	(44.2%)	27	(33.3%)	
Stable	27	(71.1%)	18	(41.9%)	45	(55.6%)	
Improved	3	(7.9%)	6	(14.0%)	9	(11.1%)	
Evolution of the role functioning score							*p* = 0.493
Worsened	17	(44.7%)	24	(55.8%)	41	(50.6%)	
Stable	11	(28.9%)	12	(27.9%)	23	(28.4%)	
Improved	10	(26.3%)	7	(16.3%)	17	(21.0%)	
Evolution of the emotional functioning score							*p* = 0.017
Worsened	5	(13.2%)	17	(39.5%)	22	(27.2%)	
Stable	20	(52.6%)	19	(44.2%)	39	(48.1%)	
improved	13	(34.2%)	7	(16.3%)	20	(24.7%)	
Evolution of the cognitive functioning score							*p* = 0.353
Worsened	9	(23.7%)	15	(34.9%)	24	(29.6%)	
Stable	16	(42.1%)	19	(44.2%)	35	(43.2%)	
Improved	13	(34.2%)	9	(20.9%)	22	(27.2%)	
Evolution of the social functioning score							*p* = 0.045
Worsened	10	(26.3%)	19	(44.2%)	29	(35.8%)	
Stable	14	(36.8%)	18	(41.9%)	32	(39.5%)	
Improved	14	(36.8%)	6	(14.0%)	20	(24.7%)	
Symptom scales							
Evolution of the fatigue score							*p* = 0.007
Worsened	16	(42.1%)	5	(11.6%)	21	(25.9%)	
Stable	7	(18.4%)	11	(25.6%)	18	(22.2%)	
Improved	15	(39.5%)	27	(62.8%)	42	(51.9%)	
Evolution of the nausea and vomiting score							*p* = 0.190
Worsened	3	(7.9%)	1	(2.3%)	4	(4.9%)	
Stable	25	(65.8%)	23	(53.5%)	48	(59.3%)	
Improved	10	(26.3%)	19	(44.2%)	29	(35.8%)	
Evolution of the pain score							*p* = 0.135
Worsened	13	(34.2%)	11	(25.6%)	24	(29.6%)	
Stable	12	(31.6%)	8	(18.6%)	20	(24.7%)	
Improved	13	(34.2%)	24	(55.8%)	37	(45.7%)	
Evolution of the dyspnea score							*p* = 0.867
Worsened	6	(15.8%)	7	(16.3%)	13	(16.0%)	
Stable	22	(57.9%)	22	(51.2%)	44	(54.3%)	
Improved	10	(26.3%)	14	(32.6%)	24	(29.6%)	
Evolution of the insomnia score							*p* = 0.003
Worsened	16	(42.1%)	4	(9.3%)	20	(24.7%)	
Stable	15	(39.5%)	26	(60.5%)	41	(50.6%)	
Improved	7	(18.4%)	13	(30.2%)	20	(24.7%)	
Evolution of the appetite loss score							*p* < 0.001
Worsened	7	(18.4%)	1	(2.3%)	8	(9.9%)	
Stable	22	(57.9%)	14	(32.6%)	36	(44.4%)	
Improved	9	(23.7%)	28	(65.1%)	37	(45.7%)	
Evolution of the constipation score							*p* = 0.111
Worsened	11	(28.9%)	7	(16.3%)	18	(22.2%)	
Stable	15	(39.5%)	27	(62.8%)	42	(51.9%)	
Improved	12	(31.6%)	9	(20.9%)	21	(25.9%)	
QL: quality of life

**Table 5:** Details of the main adverse events and SAE (overall occurrence, SAE grade 3, 4 and 5)

	A: vinorelbine		B: vinorelbine + aromatase inhibitor		All patients	
	*N* = 61		*N* = 60		*N* = 121	
Adverse event overall occurrence						
Gamma GT increase	48	(78.7%)	40	(66.7%)	88	(72.7%)
Neutropenia	20	(32.8%)	32	(53.3%)	52	(43.0%)
Febrile neutropenia	6	(9.8%)	5	(8.3%)	11	(9.1%)
Thrombopenia	7	(11.5%)	16	(26.7%)	23	(19.0%)
Arterial hypertension	43	(70.5%)	38	(63.3%)	81	(66.9%)
Lymphopenia	41	(67.2%)	39	(65.0%)	80	(66.1%)
Fatigue	43	(70.5%)	38	(63.3%)	81	(66.9%)
Nausea	32	(52.5%)	32	(53.3%)	64	(52.9%)
Vomiting	12	(19.7%)	16	(26.7%)	28	(23.1%)
Septicemia/infection	22	(36.1%)	16	(26.7%)	38	(31.4%)
Colitis	1	(1.6%)	0	(0.0%)	1	(0.8%)
Digestive obstruction	0	(0.0%)	2	(3.3%)	2	(1.7%)
Pain	37	(60.7%)	37	(61.7%)	74	(61.2%)
Central neurological complication*	18	(29.5%)	6	(0.1%)	24	(19.8%)
SAE grade 3						
Febrile neutropenia			1	(1.7%)	1	(0.8%)
Fatigue			1	(1.7%)	1	(0.8%)
Nausea	1	(1.6%)			1	(0.8%)
Vomiting	1	(1.6%)			1	(0.8%)
Septicemia/infection			2	(3.3%)	2	(1.7%)
Colitis	1	(1.6%)			1	(0.8%)
Pain			1	(1.7%)	1	(0.8%)
Central neurological complication	1	(1.6%)			1	(0.8%)
SAE grade 4						
Gamma GT increase			2	(3.3%)	2	(1.7%)
Neutropenia	2	(3.3%)			2	(1.7%)
Febrile neutropenia	5	(8.2%)	3	(5.0%)	8	(6.6%)
Thrombopenia			1	(1.7%)	1	(0.8%)
Septicemia/infection	2	(3.3%)	2	(3.3%)	4	(3.3%)
Central neurological complication	1	(1.6%)			1	(0.8%)
SAE grade 5						
Febrile neutropenia			2	(3.3%)	2	(1.7%)
Fatigue			1	(1.7%)	1	(0.8%)
Septicemia/infection			3**	(5.0%)	3	(2.5%)
Digestive obstruction			1	(1.7%)	1	(0.8%)
Central neurological complication	1	(1.6%)			1	(0.8%)

* Central neurological complication included: headache, head trauma, dizziness, tremor, balance disorder, epilepsy, aphasia, memory disorder, dysarthria, hemiplegia, cerebral venous thrombosis

** 1 septicemia/infection occurred after the end of randomized treatment

**Table 6:** Details of main adverse events and SAE (overall occurrence, SAE grade 3, 4 and 5)

Every month (before C1D1, C2D1 and C3D1):
^*^ Complete blood count with hemoglobin, WBC including neutrophils, and platelets
^*^ Liver function tests including AST, ALT, ALP, GGT, and bilirubin
^*^ Renal function including creatinine and creatinine clearance
+ Supplementary blood test every week during the first month (C1D8, C1D15, C1D21) and every 2 week during the second and third months (C2D15, C3D15):
^*^ Complete blood count with hemoglobin, WBC including neutrophils, and platelets
If no abnormal value is observed, repeat complete blood test every month
^*^ Complete blood count with hemoglobin, WBC including neutrophils, and platelets
^*^ Liver function tests including AST, ALT, ALP, GGT and bilirubin
^*^ Renal function including creatinine and creatinine clearance

*WBCs* white blood count, *AST* aspartate transaminase, *ALT *alanine transaminase, *ALP* alkaline phosphatase, *GGT* gamma-glutamyl transferase

## Abbreviations

95% CI: 95% Confidence interval
AE: Adverse event
AI: Aromatase inhibitor
CB: Clinical benefit
CHEOPS: Name of the study
CR: Complete response
ERα: Estrogen-receptor alpha
GINECO: Groupe d'Investigateurs National des Etudes des Cancers Ovariens et du sein (National Investigators Group for Ovarian and Breast Cancer Studies)
HER2−: Human epidermal growth factor receptor 2 negative
HR: Hazard ratio
HR +: Hormone receptor positive
IDMC: Independent Data Monitoring Committee
ITT: Intending to treat
MET: Maintenance endocrine therapy
NCI CTCAE: Common Terminology Criteria for Adverse Events (CTCAE) elaborated by National Cancer Institute (NCI)
OR: Objective response
OS: Overall survival
OV: Oral vinorelbine
PFS: Progression-free survival
PR: Partial response
PS: Performance status
RECIST: Response Evaluation Criteria In Solid Tumors
SAE: Serious adverse event
SBR Grade: Scarff–Bloom–Richardson grade
TTP: Time to progression
